# Low Doses of Celecoxib Stimulate Human Endometrium Growth in A Three-Dimensional Culture Model

**Published:** 2013-03-06

**Authors:** Neghin Rezavand, Mozafar Khazaei, Elham Oliapanah, Hossein Nikzad, Mohammad Rasool Khazaei

**Affiliations:** 1 Fertility and Infertility Research Center, Kermanshah University of Medical Sciences, Kermanshah, Iran; 2 Anatomical Sciences Research Center, Kashan University of Medical Sciences, Kashan, Iran

**Keywords:** Endometrium, Celecoxib, Three-Dimensional Culture, Angiogenesis

## Abstract

**Background:**

The endometrium plays a pivotal role in implantation and pregnancy. Cyclooxygenase II (COX-2) has an important function in biological processes such as cell proliferation and inflammation. Celecoxib is a selective inhibitor of COX-2 with numerous pharmacologic functions. The aim of present study is to investigate the effects of celecoxib on the human endometrium in a three-dimensional (3D) culture model.

**Materials and Methods:**

In this experimental study, normal human endometria (n=10) obtained from reproductive age women were cut into 1×1 mm sections. Endometrial explants were placed between two layers of fibrin gel. To create the fibrin gel, we poured a thin layer of fibrinogen solution [3 mg/ml in medium 199 (M199)] into each well of a 24-well culture dish and added thrombin enzyme. Endometrial fragments were placed in the center of each well and covered with a second layer of fibrinogen solution. M199 supplemented with L-glutamine, fetal bovine serum (FBS, 5%) and antibiotics were added to each well. The media in each experimental well contained either1, 10 or 50 μM of celecoxib. At the end of the study, we calculated endometrial tissue growth changes by scoring methods and determined the percentage of angiogenesis. Data were analyzed by the Kruskal-Wallis method. P<0.05 was considered significant.

**Results:**

The growth scores were as follows: control (1.37 ± 0.16), 1 μM (1.96 ± 0.28), 10 μM (2.01 ± 0.25), and 50 μM (1.17 ± 0.14) celecoxib, all of which were significantly different. The angiogenesis percentages were: 25.56 ± 6.72% (control), 31.98 ± 6.18% (1 μM), 42.67 ± 7.27% (10 μM) and 23.44 ± 4.03% (50 μM), which were not significantly different from each other.

**Conclusion:**

Lower celecoxib concentrations had stimulatory effects on the growth of normal endometrium.

## Introduction

The human endometrium or mucosal lining of the uterus is a unique, special tissue which consists of surface epithelium, glands and stroma. The endometrium undergoes intense periods of proliferation, growth and angiogenesis under the effect of sexual hormones (1, 2). This tissue plays a pivotal role in reproduction; its growth and thickness is one of determining factors of fertility (1, 3, 4).

Endometriosis is a benign lesion in the pelvis and other parts of peritoneum which is defined as the existence of endometrial glands and stroma outside of the uterus. It is a hormone-dependent disease and a cause of infertility (5). Different genetic, immunological and environmental factors are considered to be causes of endometriosis, although inflammatory factors such as prostaglandins (PG) may have a role in this disease (6). Endometriosis has been considered to be an angiogenic disease (7). Medications such as letrozole, (8) raloxifene, (9) celecoxib, (10) and statins (11) have been introduced as treatments for endometriosis.

Cyclooxygenase enzymes convert arachidonic acid to PG and exist in two main isoforms, COX- 1 and COX-2. COX-1 is a housekeeping enzyme which is found in most human tissue, whereas COX-2 is mostly located in the kidneys, brain, endothelium and female reproductive system. COX-2 is induced by pathologic stimuli such as inflammation and its upregulation has been observed in some diseases (12). There is increased COX-2 expression in eutopic and ectopic tissue of endometriosis patients (13), along with increased levels of PG in their serum and peritoneal fluid (14). Some studies have reported that inhibition of PG production resulted in decreased growth of ectopic endometrial tissue (15).

Celecoxib is a diaryl-substituted pyrazole (C_17_H_14_F_3_N_3_O_2_S). It is the newest class of nonsteriod anti-inflammatory drugs (NSAIDs) and a potent, selective inhibitor of COX-2. Celecoxib has new applications in cancer chemoprevention and gynecology (16). It is considered to be an anti-angiogenic agent (17) and apoptosis inducer (18). Celecoxib has been shown to inhibit IL6 production, colony formation, cell viability and cell migration (19).

Celecoxib has been proposed for inhibition of endometriosis lesions and VEGF secretion in endometriosis (15, 16). Thus a study of the role of the COX-2 inhibitor as a novel therapeutic modality in this disease has been proposed (18). Endometrial tissue culture in a three-dimensional (3D) fibrin matrix was introduced for endometriosis research (20) and applied for a study of the drug’s effect on the endometrium (21). However no scientific report on the effect of celecoxib on human endometria has been published. Therefore, the aim of the present work is to investigate the celecoxib effect on normal human endometria in a 3D culture model.

## Materials and Methods

In this experimental study, endometrial biopsies (n=10) were taken from reproductive age women (25-40 years) who underwent surgery for either benign myoma or diagnostic laparoscopy. The Ethics Committee of Kermanshah University of Medical Sciences accepted the work on human tissue in this study and all patients signed informed consents. All chemicals and enzymes were purchased from Sigma Company (Germany). Fetal bovine serum (FBS) was purchased from Gibco Company (Denmark). The culture method has been previously reported (20) and thoroughly discussed in our previous study (21).

The endometrial samples were in the proliferative phase. Each sample was divided into two parts, one for pathologic diagnosis and the other for tissue culture. The exclusion criteria were endometrial malignancies (cancer, hyperplasia, and polyps) and patients on hormone therapy or those who used intrauterine devices (IUD) during the previous three months. Only normal endometrium data as reported by a pathologist were chosen for final analyses.

### Endometrial tissue preparation and culture

**Fig 1:**
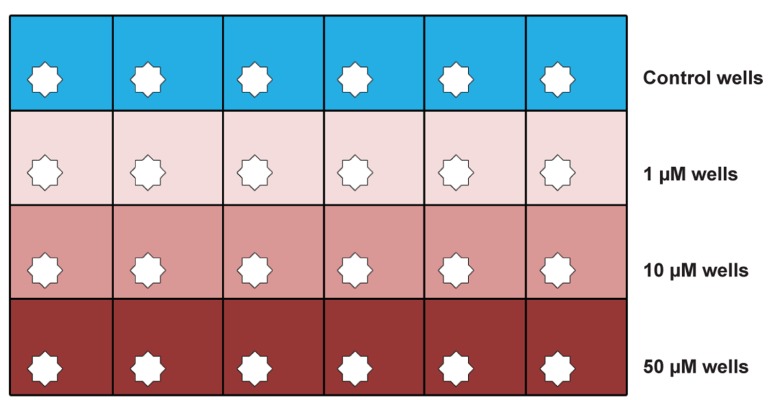
Schematic drawing of 24-well culture dish with study design for one biopsy.

Endometrial biopsies were placed in Hank’s balanced salt solution that contained amphotericin B (2.5 μg/ml) plus penicillin (50 μg/ml). Biopsies were washed and cut into small fragments (approximately 1×1 mm). We used one, 24-well culture plate (Orange Scientific) for each biopsy. Each row of the culture plate was used for either the control or one of the celecoxib doses (Fig 1).

Fibrin gel was formed by the addition of 0.5 ml/ well of fibrinogen solution (3 mg/ml in M199) to each well and mixed with 10 μL of thrombin (50 NIH U/ml in 0.15 M NaCl). Endometrial fragments were placed in the center of the wells and covered by an additional fibrinogen/thrombin solution that formed a second gel layer to hold the endometrial explants between the two clots. M199 supplemented with L-glutamine (2 mM), 5% heat-inactivated FBS, 0.1% ε-amino caproic acid, streptomycin (50 μg/ml), penicillin (50 IU/ml) and amphotericin B (2.5 μg/ml) was added to all wells. Experimental wells contained one dose of either 1 μM, 10 μM or 50 μM of celecoxib (10, 18, 22).

Tissues were cultured for 21 days and the culture media were changed every three days. The explants were cultured at 37˚C in 95% air and 5% CO_2_ in a humidified environment. On the first and the last day of the culture, explants were photographed for final comparison. At the end of the study, scoring methods were used to determine any tissue growth and morphological changes as observed by two different individuals blinded to the analyses (21) and taking into consideration cellular invasion into the fibrin matrix and capillary-like sprouting of endothelial cells.

The scoring method was: 0. no growth and tissue changes; 1. growth in less than 25% of the explant; 2. growth in 26% to 50% of the explant; 3. growth in 51% -75% of the explant and 4. growth in more than 75% of each explant. The mean of two scores was used for data analysis. We studied ten biopsies, each with a control and experimental groups. We compared the mean score of all control wells from the ten biopsies with the mean of score of the three different celecoxib doses (1, 10 and 50 μM).

The numbers of wells that showed angiogenesis were determined and percent of angiogenesis was calculated. To document endothelial cell sprouting into the fibrin matrix, we fixed the fibrin clots in 4% paraformaldehyde. Tissue processing was performed and microscopic slides immunohistochemically stained for endothelial cell marker (CD31) with anti-CD31 antibody (21).

### Statistical analyses

The scores were quantitative and with no normal distribution, thus statistical analyses were performed with the Kruskal-Wallis method using SPSS software (version 16). A p value of <0.05 was considered significant.

## Results

**Fig 2:**
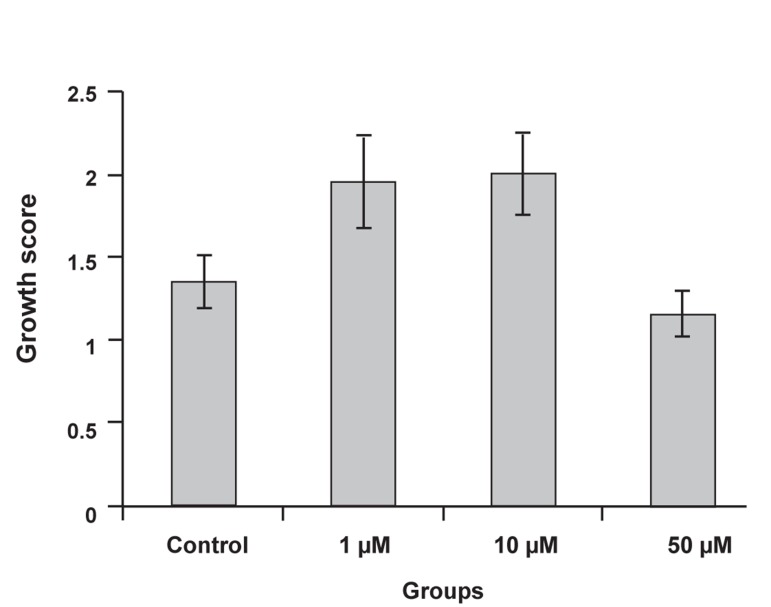
Comparison of growth score between control and experimental groups. The growth scores were higher at the 1 and 10 μM celecoxib concentrations; their difference with control groups was significant (p<0.05).

The mean growth score was 1.37 ± 0.16 for the control. The celecoxib groups had the following mean growth scores: 1.97 ± 0.28 (1 μM), 2.01 ± 0.25 (10 μM ) and 1.17 ± 0.14 (50 μM). The difference between groups was significant (p=0.03). The results showed that 1 and 10 μM concentrations of celecoxib stimulated endometrial growth and increased the growth score. The 50 μM concentration did not stimulate endometrial growth and its growth score approximated that seen with the control group (Fig 2).

**Fig 3:**
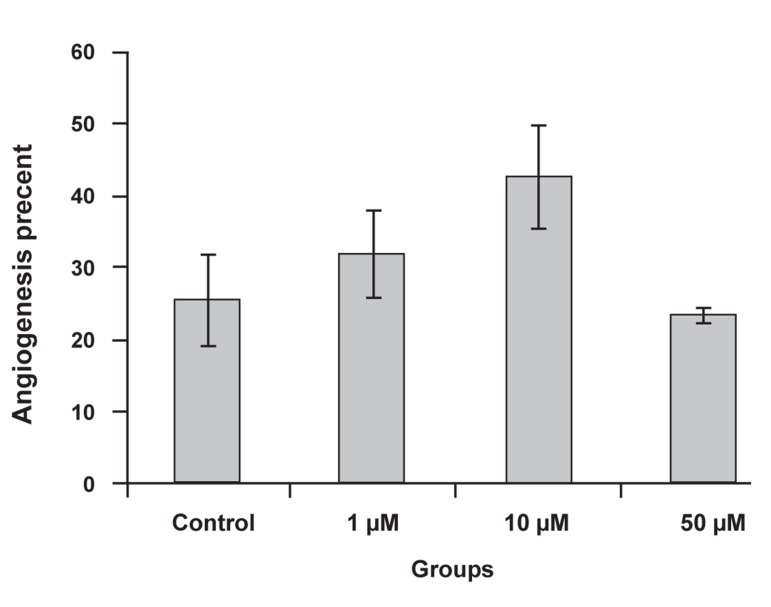
Comparison of angiogenesis percent between control and experimental groups. The higher angiogenesis percent was seen at the 10 μM celecoxib concentration.

The percent of angiogenesis observed was 25.56 ± 6.72% for the control and 31.89 ± 6.18% (1 μM), 42.67 ± 7.27% (10 μM) and 23.44 ± 4.03% (50 μM) for the celecoxib groups, which was not a significant difference. Angiogenesis was higher at the 1 and 10 μM celecoxib concentrations, which related to their growth scores (Fig 3).

**Fig 4:**
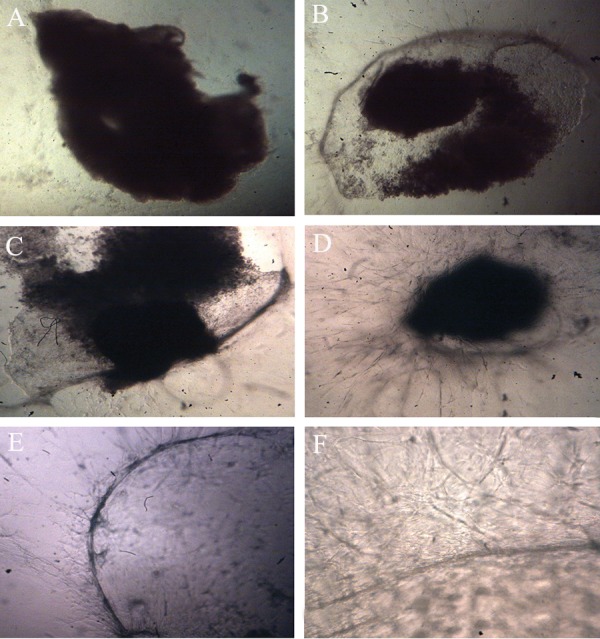
Invert microscopic photographs of endometrial fragment. (A) First day of culture showing no growth and changes in the explant (magnifications ×40). On the 21st day, cellular outgrowths were visualized (B; magnification: ×40) (C) 1 μM (×40 magnification), (D) 10 μM (magnification ×40) and (E) 50 μM( magnification ×40). (F) Angiogenesis of explant in 1 μM celecoxib at the end of the study (magnifications ×100).

**Fig 5:**
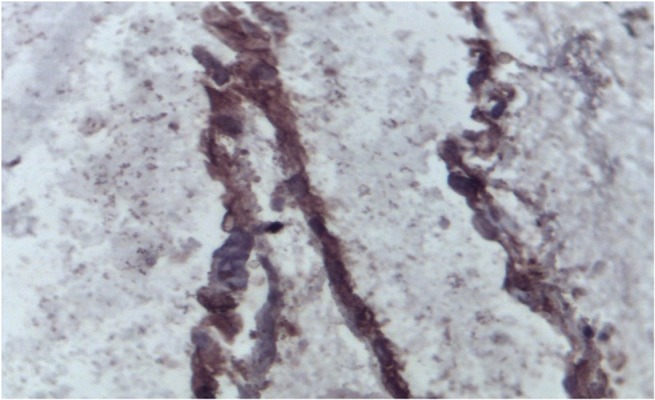
Endothelial cell projections (brown) that were immunohistochemically stained with anti-CD31 antibody.

Cellular outgrowths were visualized from the endometrial explant during the culture period. These projections consisted of epithelial, endothelial and stromal cell invasions into the fibrin matrix that were morphologically distinguishable by invert microscope (Fig 4). Endothelial cell projections were positive for anti- CD31antibody (Fig 5).

## Discussion

To our knowledge, this is the first report of the effects of celecoxib on normal human endometrium in a 3D culture model. We used three celecoxib concentrations (1, 10 and 50 μM). The 1 and 10 μM concentrations of celecoxib showed significant growth and angiogenesis stimulatory effects, however the 50 μM did not considerably affect the endometrial tissue; its growth score was lower than the control group. We observed a correlation between growth and angiogenesis in each experimental group.

The 3D culture system is a relatively new, suitable model for studying endometrial tissue culture. The fibrin matrix provides excellent extracellular matrix for stromal, epithelial and endothelial cell invasion. Gland reconstruction and endothelial cell proliferation (angiogenesis) have been visualized in this model (20, 21).

The human endometrium is a unique, dynamic and important tissue that has a central role in uterine pathophysiology. It has an intensive period of proliferation, growth, angiogenesis and remodeling (23).

Clinical disorders of the endometrium lead to a range of gynecology problems, particularly infertility (24). Endometrial growth and development is an important factor in female fertility (3). Several regimens have been introduced to improve a poor endometrium, and include estrogen therapy and low dose aspirin (25, 26). In recent years, inhibition of COX-2 has been researched in numerous studies, particularly cancer (19). Growing evidence, however, suggests that the functional significance of COX-2 is far beyond what was initially revealed (27, 28).

COX-2 is expressed in both eutopic and ectopic endometrium, although its mRNA is higher in an ectopic endometrium. COX-2 expression is directly related to malignancy (hyperplasia and cancer), thus its inhibitor can be used for treatment of inflammatory diseases. In previous reports, the effects of celecoxib on endometrial stromal cells (10), eutopic and ectopic endometrial epithelial cells (18) and endometrial tissue implanted outside of the uterus in an animal model (17) have been investigated.

Most studies have used higher concentrations of celecoxib (50 μM and more) over a short period of time (3-5 days). Here, we examined 50 μM and lower concentrations of celecoxib (1 and 10 μM) for longer culture periods (3 weeks). The growth stimulatory effect of lower celecoxib concentrations in the current study has not been considered in previous research. Higher celecoxib concentration induced apoptosis in some cell lines (29, 30).

The growth stimulatory effect of celecoxib on epithelial and endothelial cells (angiogenesis) in our study is considerable and it can be used to improve endometrial thickness in an ART cycle. The angiogenic effect of lower celecoxib doses (1 and 10 μM) in the present study contrasts the antiangiogenic effects of a COX-2 inhibitor, as has been previously reported (22). An explanation for this contradiction is the higher (50 μM and more) concentration of celecoxib studied in the previous research. Additional studies are necessary in order to define the mechanism of stimulating endometrial tissue growth and the angiogenic effect of celecoxib on the human endometrium.

## Conclusion

In this 3D culture model, the lower celecoxib concentrations show stimulatory effects on normal human endometrium, whereas the higher celecoxib concentration had inhibited endometrial growth.
